# Treatment of benign lesions of the proximal femur in young pediatric patients using adult 3.5 mm proximal humerus locking plates

**DOI:** 10.3389/fonc.2025.1576306

**Published:** 2025-10-03

**Authors:** Zhiqiang Zhao, Xin Liu, Xianbiao Xie, Changye Zou, Junqiang Yin, Haomiao Li, Jingnan Shen

**Affiliations:** ^1^ Department of Musculoskeletal Oncology, The First Affiliated Hospital of Sun Yat-sen University, Guangzhou, China; ^2^ Third Affiliated Hospital, Southern Medical University, Guangzhou, China

**Keywords:** proximal femur, benign tumor, pediatric, pathological fracture, proximal humerus locking plate (PHLP)

## Abstract

This study aimed to assess the efficacy of using adult 3.5 mm proximal humerus locking plates (PHLPs) for treating benign proximal femur lesions in skeletally immature pediatric patients. Between 2011 and 2021, 22 skeletally immature pediatric patients were treated with adult 3.5 mm PHLPs for benign lesions of the proximal femur. During this period, indications for the procedure included lesions involving bone cyst (BC), aneurysmal bone cyst (ABC), and fibrous dysplasia (FD) of the proximal femur, resulting in impending or actual pathologic fractures. The mean follow-up time for the entire group of patients (n=22) was 57 months (range, 27–118 months). A visual analog scale (VAS) scoring system was used for patients with pathological fractures to assess postoperative pain relief. Functional outcomes were evaluated using the Musculoskeletal Tumor Society (MSTS) lower extremity scoring system. Postoperatively, the mean total MSTS score (measured at the most recent follow-up) was 29.41 (95% CI, 29.11 to 29.70) for the entire group of patients. The mean pain relief for patients with actual pathological fractures assessed by VAS was 4.88 (95% CI, 4,30 to 5.45). One patient with ABC and two patients with FD experienced recurrences, necessitating a second surgery at 22, 10, and 15 months post-initial surgery, respectively. Following the second surgery, the locking plates in the patients with ABC and one FD case were removed 30 and 12 months after the initial surgery. The third FD patient, who did not have a pathological fracture, retained the locking plate as of the latest follow-up. Only one patient had delayed wound healing after the initial surgery, and the others had no other complications. 18 patients had their locking plates removed at a mean time of 23 months (95% CI, 18 to 28). In conclusion, treatment of benign lesions of the proximal femur in young pediatric patients with adult 3.5 mm PHLPs provides good functional outcomes and radiological results.

## Introduction

The proximal femur is one of the most common locations for benign bone lesions in young children and adolescents. Benign bone lesions, including bone cysts (BCs), aneurysmal bone cysts (ABCs), and fibrous dysplasia (FD), are frequently found in this region (1, 2). Although typically small and asymptomatic, these lesions consistently lead to pain, claudication, or even an inability to walk, often accompanied by imminent or actual moderate to large pathological fractures.

Pediatric proximal femur fractures, especially subtrochanteric fractures secondary to benign lesions, are a great challenge for orthopedic surgeons because of the high risk of postoperative recurrence, avascular necrosis (AVN) of the proximal femur, malunion, nonunion, and leg length discrepancy (LLD) (3). As a weight-bearing limb, surgical intervention should adequately eradicate the tumor lesion to get local control of the disease and reconstruct the anatomical integrity of the affected limb (3, 4). The selection of treatment strategy primarily depends on two factors: the size of the bony defect following curettage of the lesion and the patient’s age. There is limited literature on treatment strategies for benign tumors of the proximal femur, particularly subtrochanteric benign tumors, in pediatric patients (5–8). In addition, in treating pathological fractures of the proximal femur, maintaining stability is important for fracture healing, early ambulation of the lower limb, maintenance of lower limb force lines, and reduction of complications, such as joint stiffness and wasted bone. However, several conventional locking plates or bone graft external fixations are unsuitable for mechanical stabilization in skeletally immature pediatric patients, especially those with small sizes and significant bony defects. To our knowledge, two articles have reported a total of two cases involving the off-label use of an adult proximal humerus locking plate (PHLP) in patients with bone tumors (9, 10).

The purpose of this study was to assess the application of proximal humeral locking plates (PHLPs) for reconstruction in skeletally immature pediatric patients with benign proximal femoral tumors, and to examine functional outcomes, complications, and bone healing after surgery.

## Materials and methods

### Patient population

**Figure 1:**
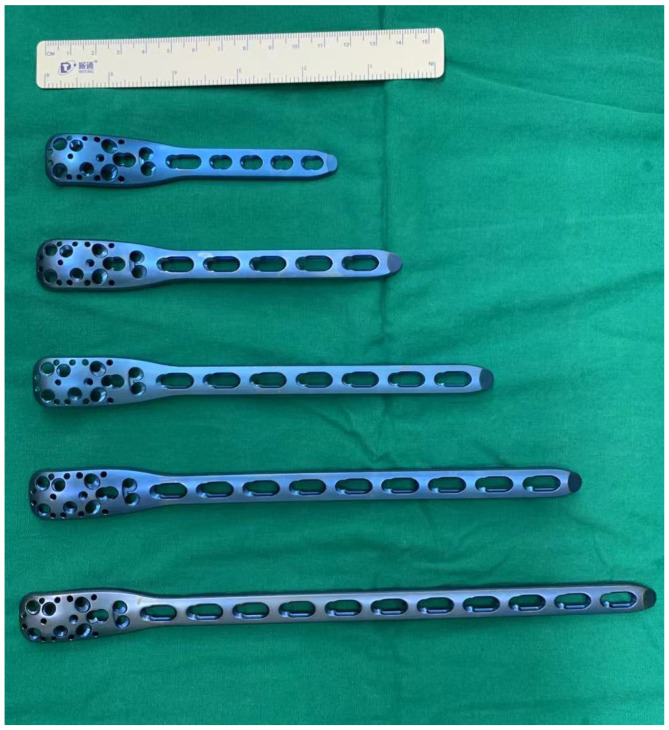
Different sizes of adult 3.5 mm proximal humerus locking plates were used in this study.

Between 2011 and 2021, 22 skeletally immature patients with benign lesions of the proximal femur underwent stabilization using 3.5 mm PHLPs (an off-label use) for impending or established pathologic fractures (**Figure 1**). This study received approval from the Ethics Committee of the First Affiliated Hospital of Sun Yat-sen University and informed consent was obtained from all legal guardians. Prior to surgery, surgeons explicitly informed the parents about the potential risks associated with the implant to ensure ethical transparency and mitigate liability.

#### Inclusion criteria

Inclusion criteria comprised benign lesions of BCs, ABCs, and FD involving the proximal femur, particularly the subtrochanteric femur, resulting in impending or actual pathologic fractures. The impending fractures were classified as painful lesions with over 50% cortical erosion (11).

#### Exclusion criteria

Exclusion criteria included massive proximal femoral destruction (inadequate fixation purchase), active infection, or inability to comply with protected weight-bearing protocols.

The duration of follow-up for patients (n=22) in this study ranged from 27 to 118 months (**Table 1**). Patients included 17 males aged 3 to 11 years and 5 females aged 4 to 11 years (**Table 1**). Patients with impending fractures (n = 6) had painful lesions with cortical erosion. Patients with actual pathologic fractures (n = 16) had mild-to-moderate displacement and angulation. Imaging and needle biopsy or intraoperative frozen biopsy were also taken to determine the types of lesions.

**Table 1:** Patient Characteristics (N=22).

Age* (year)	8 (range 3 to11)
Gender	
Female	5 (22.7%)
male	17 (77.3%)
Diagnosis	
FD	11 (50.0%)
ABC	9 (40.9%)
BC	2 (9.1%)
Site	
L	13 (59.1%)
R	9 (40.9%)
Affected part	
STRO	13 (59.1%)
FN+ITRO	4 (18.2%)
ITRO	5 (22.7%)
Fracture	
Actual	16 (72.7%)
Impending	6 (27.3%)
Treatment	CUR+BG+P&S

*The values are given as mean (range min to max).

BC, bone cyst; FD, fibrous dysplasia; ABC, aneurysmal bone cyst; R, right; L, left; STRO, Subtrochanteric; FN, femoral neck; ITRO, Intertrochanteric; CUR, curettage; BG, bone graft; P&S, internal fixation with locking plate and screws.

#### Surgical procedures

**Figure 2:**
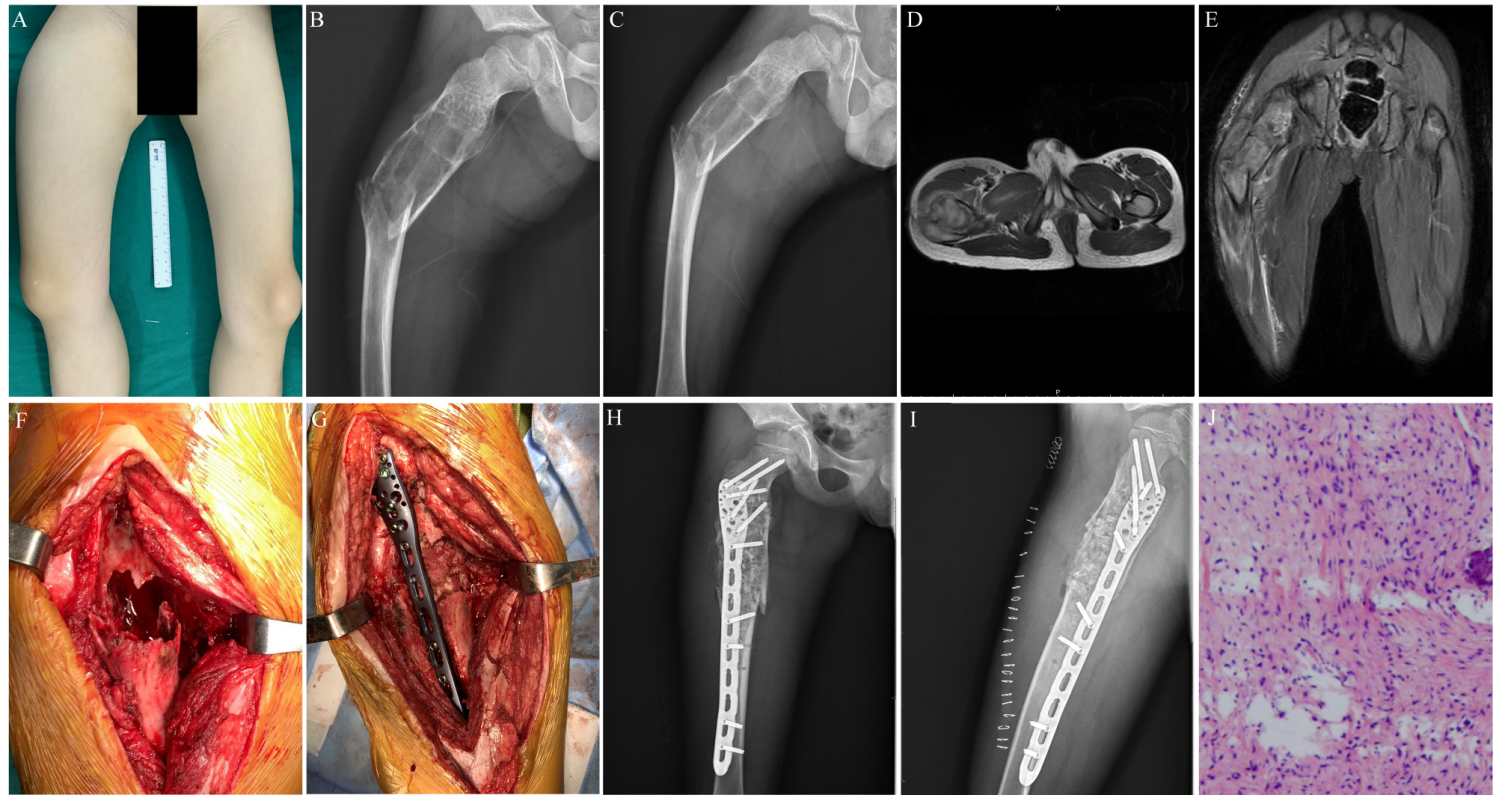
Preoperative, postoperative, and operative images of a 7-year-old male patient with a pathological fracture of the proximal end of the right femur. **(A)** A general image of the patient before surgery. **(B)** Preoperative lateral x-ray image of the patient. **(C)** Preoperative posteroanterior x-ray image of the patient. **(D)** Preoperative transverse MR image of the patient. **(E)** Preoperative coronal MR image of the patient. **(F)** Enlarged cortical window with adequate scraping of the lesion. **(G)** Bone grafting, internal fixation with a suitable adult 3.5 mm proximal humerus locking plate, and gross fracture reduction. **(H)** Postoperative x-ray image of the patient. **(I)** Postoperative lateral x-ray image of the patient. **(J)** Postoperative histopathological diagnosis of the patient.

**Figure 3:**
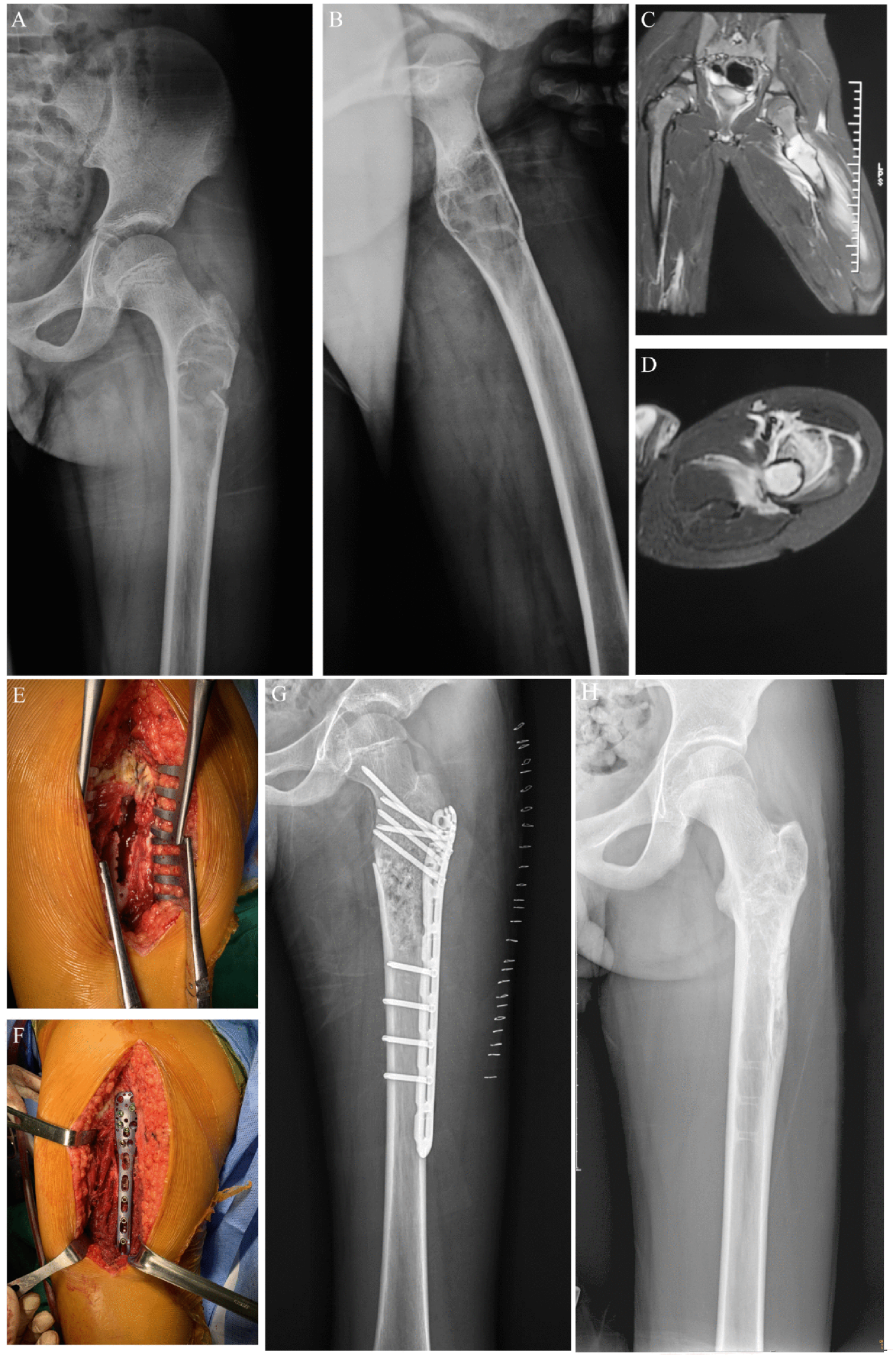
Preoperative, postoperative, and operative images of a pathological fracture of the proximal end of the left femur in an 11-year-old male patient. **(A)** Preoperative posterior radiographic image of the patient. **(B)** Preoperative lateral x-ray image of the patient. **(C)** Preoperative coronal MR image of the patient. **(D)** Preoperative transverse MR image of the patient. **(E)** Enlarged cortical window with adequate scraping of the lesion. **(F)** Bone grafting, internal fixation with a suitable adult 3.5 mm proximal humerus locking plate, and gross fracture reduction. **(G)** Postoperative x-ray image of the patient. **(H)** X-ray image of the patient after the removal of the metal plate.

The surgical plan included an intraoperative frozen section, biopsy scraping, bone grafting, and mechanical stabilization. The patients were placed laterally on the operating table, and the proximal femur was laterally sectioned under the guidance of an intraoperative x-ray. Of these patients, some (n = 14) were diagnosed with benign tumors by preoperative needle biopsy, and others (n = 8) were analyzed by cryobiopsy during surgery. Pathologic tissue was taken from the fracture end for intraoperative frozen biopsy in patients with pathologic fractures. After pathology reported no malignant cells, the cortical window was enlarged (for patients with impending pathological fractures) or coarsely reduced bone fragments (for patients with actual pathological fractures) to allow good lesion scraping. During surgery, iodine tincture was added to the tumor cavity, and electrosurgical ablation and high-speed grinding ablation were performed to inactivate the tumor lesion adequately. After good scraping and inactivation of the lesion, we lightly shaped adult 3.5 mm PHLPs (PHILOS; Synthes) with drill guides (**Figure 1**) to ensure a close fit with the patient’s anatomy, specifically the greater trochanter. For patients with impending pathological fractures, we filled the bony defect with demineralized bone matrix and bone morphogenetic protein-2 (BMP-2). In contrast, for patients with actual pathological fractures, reduction, and initial fixation were performed before filling. We chose a long locking plate with additional distal screws to maximize stability and then fixed the plate distally to the bone. We selected the most appropriate several screw holes out of the nine available at the proximal end of the plate to place multiple 3.5 mm locking screws into the patient’s femoral neck. Utilizing intraoperative X-rays, we ensured optimal engagement of the screws and carefully avoided obstructing the proximal femoral epiphysis (**Table 2**). We then fixed the distal end of the plate with suitable 3.5 mm cortical screws and performed conventional closure (**Figures 2**, **3**).

**Table 2:** Patient Outcomes (N=22).

Follow-up period (months)*	57 (range 27 to 118)
MSTS Score	29.41 (95% CI 29.1 to 29.70)
VAS Score (PRE)	6.73 (95% CI 5.88 to 7.57)
VAS Score (POST)	2.68 (95% CI 2.36 to 3.00)
VAS Score (PRE-POST)	4.05 (95% CI 3.29 to 4.80)
Number of LSFN	3 (95% CI 2 to 4)
Recurrence rate	13.6% (95% CI -1.9% to 29.2%)
Bone union rate	100% (95% CI 100% to 100%)
Complication rate	4.6% (95% CI -4.9% to 14.0%)

*The values are given as mean (range min to max).

MSTS, the Musculoskeletal Tumor Society Score; VAS, Visual Analogue Scale; PRE, Pre-operation; POST, Post-operation; LSFN, Locking Screws placed into the Femoral Neck.

### Postoperative patient management

After surgery, the patient received two weeks of oral prednisone (10–20 mg per day) to reduce the risk of postoperative rejection reactions and intravenous antibiotics until the drainage tube was removed. Patients were hospitalized at our institution for approximately five to seven days postoperatively and then discharged to any of our rehabilitation centers. Patients with impending fractures were allowed to bear weight on the third postoperative day, while those with actual pathological fractures began weight-bearing approximately one month later. All patients were instructed to follow a hip prophylaxis protocol for two months, which involved assistive devices to ensure a gradual and safe return to weight-bearing, thus protecting the hip joint during recovery. The Visual Analog Scale (VAS) was utilized to assess pain relief. Preoperative VAS scores were recorded to establish a baseline. Postoperatively, the VAS scores were reassessed at the time of the patient’s first weight-bearing experience, aligning with the weight-bearing initiation timing for each fracture type. The VAS scores ranged from 0 (no pain) to 10 (worst pain).

### Assessment of functional outcomes

Functional outcomes were assessed during the follow-up period according to the Musculoskeletal Tumor Society (MSTS) scoring system for lower extremities. The MSTS scoring system for lower extremities includes pain, function, emotional acceptance, support, walking, and gait (12). Scores, complications, and bone healing were determined by direct history taking, physical examination, imaging examination, and review of institutional databases.

### Statistical analysis

The SPSS statistical package program (SPSS version 24.0; SPSS Inc., Chicago, Illinois, USA) was used for statistical analysis. The Chi-Squared (χ2) test was used to analyze categorical data, and the Student t-test was used to analyze continuous data. The data is expressed as a mean (95% CI) or as a percentage (%). *P* < 0.05 was considered significantly different.

## Results

**Figure 4:**
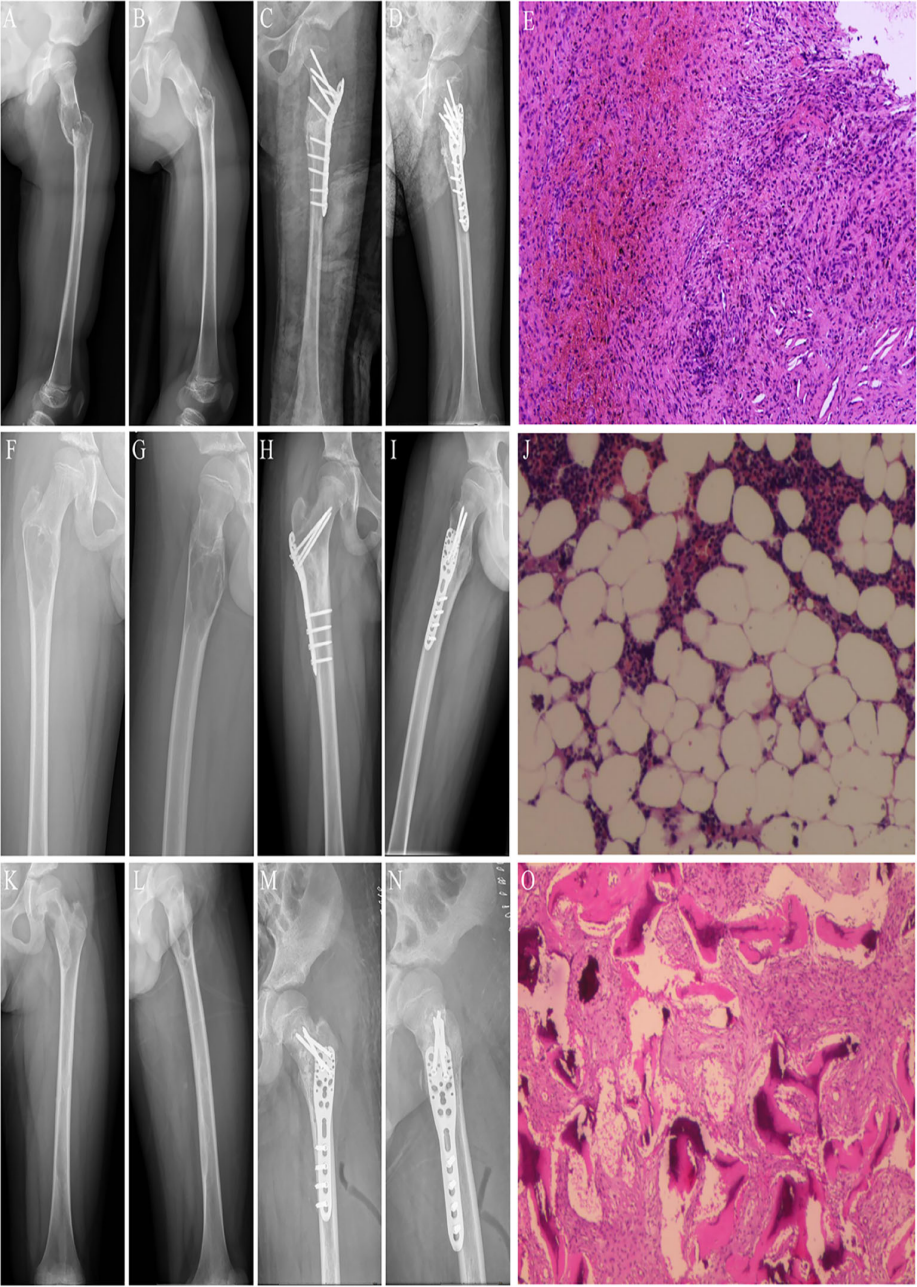
The three types of benign lesions were treated in this study. **(A)** Preoperative posteroanterior x-ray image of a patient with a pathological fracture of the left proximal femur caused by BC. **(B)** Preoperative lateral x-ray image of a patient with a pathological fracture of the left proximal femur caused by BC. **(C)** Postoperative posteroanterior x-ray image of a patient with BC of the left proximal femur. **(D)** Postoperative lateral x-ray image of a patient with BC of the left proximal femur. **(E)** Postoperative pathologic image of BC. **(F)** Preoperative posteroanterior x-ray image of a patient with a pathologic fracture of the left proximal femur caused by ABC. **(G)** Preoperative lateral x-ray image of a patient with a pathological fracture of the left proximal femur caused by ABC. **(H)** Postoperative posteroanterior X-ray image of one patient with ABC of the left proximal femur. **(I)** Postoperative lateral x-ray image of a patient with ABC of the left proximal femur. **(J)** Postoperative pathological image of ABC. **(K)** Preoperative posteroanterior x-ray image of one patient with a pathological fracture of the left proximal femur caused by FD. **(L)** Preoperative lateral x-ray image of a patient with a pathological fracture of the left proximal femur caused by FD. **(M)** Postoperative x-ray image of a patient with FD of the left proximal femur. **(N)** Postoperative lateral x-ray image of a patient with FD of the left proximal femur. **(O)** Postoperative pathologic image of FD.

**Figure 5:**
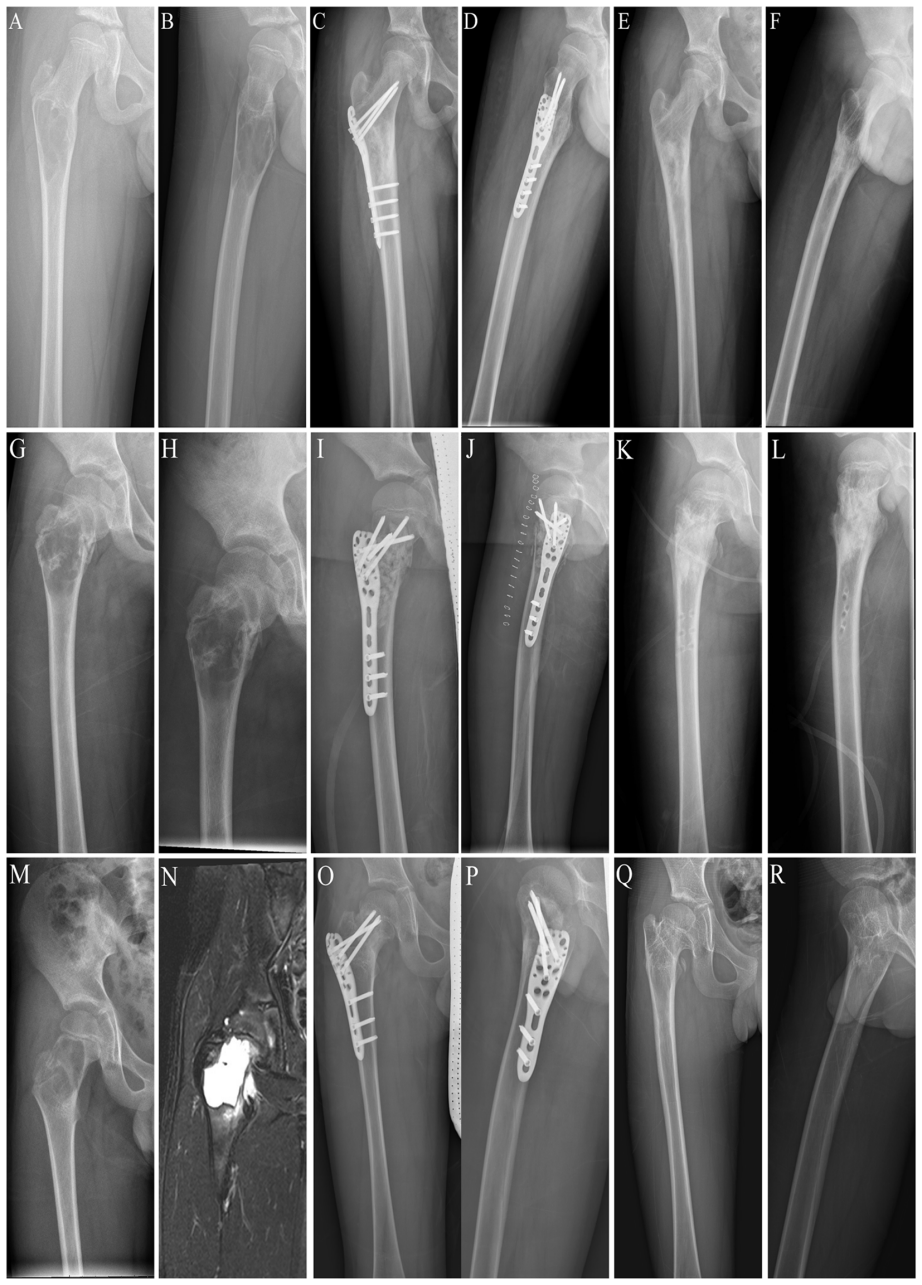
Three different locations of benign lesions of the proximal femur. **(A)** Preoperative posteroanterior x-ray of a subtrochanteric lesion. **(B)** Preoperative lateral x-ray image of a subtrochanteric lesion. **(C)** Postoperative posteroanterior x-ray image of the internal fixation for a subtrochanteric lesion. **(D)** Postoperative lateral x-ray image of the internal fixation for a subtrochanteric lesion. **(E)** Posteroanterior x-ray image after removing the locking plate and screws from the subtrochanteric lesion. **(F)** Lateral x-ray image after removing the locking plate and screws from the subtrochanteric lesion. **(G)** Preoperative posteroanterior x-ray image of an intertrochanteric lesion. **(H)** Preoperative lateral x-ray image of an intertrochanteric lesion. **(I)** Postoperative posteroanterior x-ray image of internal fixation for an intertrochanteric lesion. **(J)** Postoperative lateral x-ray image of the internal fixation for an intertrochanteric lesion. **(K)** Posteroanterior x-ray image after removal of the locking plate and screws from the intertrochanteric lesion. **(L)** Lateral x-ray image after removing the locking plate and screws from the intertrochanteric lesion. **(M)** Preoperative posterior x-ray images of the intertrochanteric part and the femoral neck lesion. **(N)** Preoperative lateral x-ray image of the intertrochanteric segment and the femoral neck lesion. **(O)** Postoperative posteroanterior x-ray image of internal fixation for the intertrochanteric part and the femoral neck lesion. **(P)** Postoperative lateral x-ray image of internal fixation for the intertrochanteric part and the femoral neck lesion. **(Q)** Posteroanterior x-ray image of the intertrochanteric part and femoral neck lesion after removing the locking plate and screws. **(R)** Lateral x-ray image after removing the locking plate and screws from the intertrochanteric and femoral neck lesions.

Patients with benign lesions of the proximal femur comprised three types of lesions, including BC (n = 2), ABC (n = 9), and FD (n = 11) (**Table 1**) (**Figure 4**). The postoperative pathology of all cases was consistent with the preoperative or intraoperative frozen biopsy findings, and none was misdiagnosed. Based on the affected part of the femur, patients were divided into three groups, including the subtrochanteric part (n = 13), intertrochanteric part (n = 5), and both intertrochanteric part and femoral neck (n = 4) (**Figure 5**). For each patient, a mean of 3 (95% CI, 2 to 4) locking screws were placed into the femoral neck to secure the locking plate at the proximal end of the femur. In contrast to conventional bone plates, no screws passed through the epiphysis or into the femoral head.

For patients with at least two years of follow-up (n = 22), full weight-bearing and mobilization were possible without pain and limping at the most recent follow-up visit. The mean VAS score measured preoperatively for the entire group of patients was 6.73 (95% CI, 5.88 to 7.57), and the mean VAS score calculated on the first day of weight-bearing was 2.68 (95% CI, 2.36 to 3.00). For patients with actual pathological fractures, the mean VAS scores measured preoperatively and on the first day of weight-bearing were 7.69 (95% CI, 7.08 to 8.29) and 2.81 (95% CI, 2.41 to 3.21), respectively. The mean pain relief in patients with actual pathological fractures assessed by VAS scores was 4.88 (95% CI, 4,30 to 5.45) (P<0.05). Several patients felt some discomfort after surgery and could not move freely as they did before surgery because of the fear of re-fracture of the affected limbs. However, these patients had no significant pain or motor impairment during our follow-up period. Early psychological fears resolved spontaneously and did not impact final functional scoring. The mean total MSTS score measured at the most recent follow-up for the entire group of patients was 29.41 (95% CI, 29.11 to 29.70) (**Table 2**).

Of the 22 patients who underwent initial surgery, 19 (86.4%) achieved complete clinical recovery. One patient with ABC and two patients with FD experienced recurrences, necessitating a second surgery at 22, 10, and 15 months post-initial surgery, respectively. Following the second surgery, the locking plates in the patients with ABC and one FD case were removed 30 and 12 months after the initial surgery. The third FD patient, who did not have a pathological fracture, retained the locking plate as of the latest follow-up. (**Table 2**).

Only one (4.5%) patient experienced delayed wound healing after the initial surgery, and the others had no other complications, including deep infection, fractures of plate or screw, LLD, and avascular necrosis of the proximal femoral epiphysis. According to the latest follow-up data, 22 patients (with over two years of follow-up) did not develop necrosis or collapse of the femoral head, and the rate of bone healing in these patients was 100%. 18 patients had their locking plates removed for a mean of 23 months (95% CI, 18 to 28).

## Discussion

The proximal femur is a general site for benign bone lesions in children. Most of these lesions are cystic, cyst-like, with bony defects and abnormal bone development, and often extend from the subtrochanteric to the femoral neck (5, 13, 14). In the present study, only 13 patients were affected by the subtrochanteric portion, and five patients were affected by the intertrochanteric part. Four patients were affected by both the intertrochanteric part and the femoral neck. Small, silent, and asymptomatic lesions rarely require treatment. However, larger lesions that progressively cause pain, affect gait, or lead to walking difficulties require timely surgical treatment, especially when fractures are likely or have occurred.

Even in patients with benign lesions, pathologic fractures of the proximal femur that are imminent or have occurred can cause moderate-to-severe pain and loss of walking function. Effective surgical intervention includes good scraping, bone grafting, and stable fixation. In young pediatric patients with pathologic fractures of the proximal femur, the primary goals of fixation are to reduce pain, maintain fracture stability, maintain the lower limb force line, and promote bone graft healing. In addition, prevention of re-fracture and early weight-bearing is important in young pediatric patients. In the present study, the mean pain relief in patients with actual pathological fractures assessed by VAS score was 4.88 (95% CI, 4,30 to 5.45) (P<0.05). The mean total MSTS score (measured during the second-year postoperative follow-up) for the entire group of patients was 29.41 (95% CI, 29.11 to 29.70) (**Table 2**).

Several surgical treatment options in skeletally mature patients include external fixator, elastic nails, proximal femoral nail counter-rotation, dynamic hip screws, intramedullary nails, and plates with screws (13, 15, 16). However, getting adequate fixation of the proximal femur in skeletally immature pediatric patients is also challenging because of the nonunion of the proximal femoral epiphysis and the high risk of AVN (7, 8). The Pediatric Hip Plate (PHP) is an excellent alternative for internal fixation strategies. However, its use is associated with certain challenges due to the unique anatomy of the pediatric femoral neck, which increases the risk of damaging the growth plate (17). Given that, we opted for the adult proximal humerus plate due to its slim profile and ease of shaping, which made it suitable for the patient’s anatomy. This plate’s design provided us with the flexibility needed for the procedure, despite not being specifically designed for the femur. To mitigate the increased stiffness of adult PHLPs compared to pediatric-specific implants, we adopted several strategies: limiting proximal screw usage (mean: 3 screws) to reduce stress shielding, applying demineralized bone matrix and BMP-2 to promote osteointegration, and utilizing intraoperative X-rays to avoid growth plate injury. For lesions in the middle of the femur, elastic stable intramedullary nailing (ESIN) combined with bone grafting is also a good option for pediatric patients (18). However, for lesions involving the proximal femur (e.g. femoral trochanter or femoral neck), especially in patients with cortical bone destruction or defects, ESIN is difficult to provide adequate, stable fixation. In addition, heavy pediatric patients do not allow early weight-bearing, and insertion of an ESIN may disrupt or contaminate the normal medullary cavity of the distal femur. In addition, rigid nailing increases the risk of AVN of the femoral head in pediatric patients (19, 20). This study used an adult 3.5 mm PHLP for internal fixation on the proximal femur in pediatric patients. To our knowledge, two case reports have shown similar success with this type of fixation. One was the treatment of a pediatric subtrochanteric nonunion, and the other was repairing an acute pathologic subtrochanteric fracture of the femur caused by BC (9, 10). In our study, 22 pediatric patients with proximal femoral lesions were treated with adult 3.5 mm PHLPs, the most extensive study reported.

There are several advantages to this surgical approach. First, the multiple lengths of locking plates provide more stable fixation and an optimal environment for bone healing. Second, the multiple locking holes at the proximal end of the humeral locking plate allow us to select the optimal locking hole and locking angle for the specific situation during the procedure, which is the most significant advantage over other devices. In addition, the size of the locking plate and the diameter of the locking screws are suitable for proximal femoral fixation in young pediatric patients.

Of the 22 patients who underwent initial surgery, 19 (86.4%) achieved complete clinical recovery. The three additional patients who experienced recurrences also achieved bone healing following their second surgeries. We acknowledge that the present study did not quantitatively assess lesion size or resection margins, which is a limitation. However, existing literature provides relevant context: for example, Basarr et al. (21) reported no significant association between recurrence and either physeal involvement or lesion size in pediatric benign bone lesions. The functional outcomes in the three patients were acceptable. Therefore, these implants can be alternatives to traditional surgical strategies, such as external fixation or intramedullary devices.

Complications in this study were uncommon, although this would have to be major surgery by any definition. In this series, only one patient had delayed wound healing after the initial surgery (curettage + bone grafting + internal fixation) (**Figures 2**, **3**). Studies on the treatment of pediatric pathological fractures in the proximal femur are limited. Malkawi et al. (22) showed that three (25%) complications occurred, including nonunion and stress fractures. Roposh et al. (8) showed that one of 12 pediatric patients with BC of the proximal femur developed coxa vara deformity after retrograding flexible nailing.

After surgery for pathological fractures in the proximal femur, nonunion is a catastrophic complication in pediatric patients (7, 8). Pediatric patients should be promptly corrected once bone nonunion or malunion is detected during follow-up (18). Among the patients in this study, no patient developed bone nonunion or malunion.

This study has several limitations. First, the absence of a control group treated with alternative fixation methods (e.g., ESIN or pediatric-specific plates) restricts direct comparative conclusions. Second, the small cohort (n=22) may limit the generalizability of our findings, particularly for rare lesions like BC or FD. Finally, the variability in follow-up duration (27–118 months) could affect outcome consistency; however, our analysis showed no significant correlation between follow-up time and MSTS/VAS scores (p>0.05). Future randomized controlled trials (RCTs) comparing PHLPs to pediatric-specific implants, as well as multi-center studies with larger samples and standardized follow-ups, are warranted to validate these findings.

## Conclusions

Treatment with an adult 3.5 mm PHLP provides excellent functional results and radiographic outcomes. The role of this fixation method is to provide adequate support and acceptable functional outcomes for pediatric patients in the early postoperative period.

## Data Availability

The original contributions presented in the study are included in the article/supplementary material. Further inquiries can be directed to the corresponding author.

## References

[1] Dormans JP Pill SG. Fractures through bone cysts: unicameral bone cysts, aneurysmal bone cysts, fibrous cortical defects, and nonossifying fibromas. Instr Course Lect. (2002) 51:457–67., PMID: 12064135

[2] Ortiz EJ Isler MH Navia JE Canosa R. Pathologic fractures in children. Clin Orthop Relat Res. (2005) 432):116–26. doi: 10.1097/01.blo.0000155375.88317.6c, PMID: 15738811

[3] Saraph V Linhart WE. Modern treatment of pathological fractures in children. Injury. (2005) 36 Suppl 1:A64–74. doi: 10.1016/j.injury.2004.12.015, PMID: 15652939

[4] Wai EK Davis AM Griffin A Bell RS Wunder JS. Pathologic fractures of the proximal femur secondary to benign bone tumors. Clin Orthop Relat Res. (2001) 393):279–86. doi: 10.1097/00003086-200112000-00032, PMID: 11764360

[5] Vigler M Weigl D Schwarz M Ben-Itzhak I Salai M Bar-On E. Subtrochanteric femoral fractures due to simple bone cysts in children. J Pediatr Orthop B. (2006) 15:439–42. doi: 10.1097/01.bpb.0000228394.47431.d7, PMID: 17001253

[6] Erol B Pill SG Guttenberg ME Meyer JS Dormans JP. Pathologic hip fracture in a 4-year-old boy. Clin Orthop Relat Res. (2002) 403):264–73. doi: 10.1097/00003086-200210000-00038, PMID: 12360036

[7] Sung AD Anderson ME Zurakowski D Hornicek FJ Gebhardt MC. Unicameral bone cyst: A retrospective study of three surgical treatments. Clin Orthop Relat Res. (2008) 466:2519–26. doi: 10.1007/s11999-008-0407-0, PMID: 18679761 PMC2584314

[8] Roposch A Saraph V Linhart WE. Treatment of femoral neck and trochanteric simple bone cysts. Arch Orthop Trauma Surg. (2004) 124:437–42. doi: 10.1007/s00402-004-0702-5, PMID: 15205988

[9] Newbury AJ Aurigemma P Kraus M Most M. The repair of an acute pathological subtrochanteric femur fracture using an adult 3.5-mm proximal humerus locking plate in an adolescent patient: A case report. JBJS Case Connect. (2020) 10:e0491. doi: 10.2106/jbjs.Cc.19.00491, PMID: 32649111

[10] Cortes LE Triana M Vallejo F Slongo TF Streubel PN. Adult proximal humerus locking plate for the treatment of a pediatric subtrochanteric femoral nonunion: A case report. J Orthop Trauma. (2011) 25:e63–7. doi: 10.1097/BOT.0b013e3181f8d9c3, PMID: 21577158

[11] Peterson JR Decilveo AP O'Connor IT Golub I Wittig JC. What are the functional results and complications with long stem hemiarthroplasty in patients with metastases to the proximal femur? Clin Orthop Relat Res. (2017) 475:745–56. doi: 10.1007/s11999-016-4810-7, PMID: 27052019 PMC5289173

[12] Enneking WF Dunham W Gebhardt MC Malawar M Pritchard DJ. A system for the functional evaluation of reconstructive procedures after surgical treatment of tumors of the musculoskeletal system. Clin Orthop Relat Res. (1993) 286):241–6. doi: 10.1097/00003086-199301000-00035 8425352

[13] Erol B Topkar MO Aydemir AN Okay E Caliskan E Sofulu O. A treatment strategy for proximal femoral benign bone lesions in children and recommended surgical procedures: retrospective analysis of 62 patients. Arch Orthop Trauma Surg. (2016) 136:1051–61. doi: 10.1007/s00402-016-2486-9, PMID: 27317344

[14] Stephenson RB London MD Hankin FM Kaufer H. Fibrous dysplasia. An analysis of options for treatment. J Bone Joint Surg Am. (1987) 69:400–9. doi: 10.2106/00004623-198769030-00012, PMID: 3546323

[15] Fang X Liu H Lang Y Xiong Y Duan H. Fibrous dysplasia of bone: surgical management options and outcomes of 22 cases. Mol Clin Oncol. (2018) 9:98–103. doi: 10.3892/mco.2018.1636, PMID: 29977545 PMC6031034

[16] Kushare IV Colo D Bakhshi H Dormans JP. Fibrous dysplasia of the proximal femur: surgical management options and outcomes. J Child Orthop. (2014) 8:505–11. doi: 10.1007/s11832-014-0625-9, PMID: 25409925 PMC4252268

[17] Zheng P Yao Q Xu P Wang L. Application of computer-aided design and 3d-printed navigation template in locking compression pediatric hip plate(Tμ) placement for pediatric hip disease. Int J Comput Assist Radiol Surg. (2017) 12:865–71. doi: 10.1007/s11548-017-1535-3, PMID: 28190127

[18] Li J Ze R Rai S Tang X Liu R Hong P. Is elastic stable intramedullary nail a good choice for pathological fractures of the proximal femur due to simple bone cyst in pediatric population? Med (Baltimore). (2020) 99:e22364. doi: 10.1097/md.0000000000022364, PMID: 32991454 PMC7523860

[19] Pombo MW Shilt JS. The definition and treatment of pediatric subtrochanteric femur fractures with titanium elastic nails. J Pediatr Orthop. (2006) 26:364–70. doi: 10.1097/01.bpo.0000203005.50906.41, PMID: 16670550

[20] Jarvis J Davidson D Letts M. Management of subtrochanteric fractures in skeletally immature adolescents. J Trauma. (2006) 60:613–9. doi: 10.1097/01.ta.0000197606.63124.9e, PMID: 16531863

[21] Basarr K Piskin A Güçlü B Yldz Y Sagglk Y. Aneurysmal bone cyst recurrence in children: A review of 56 patients. J Pediatr Orthopaedics. (2007) 27:938–43. doi: 10.1097/bpo.0b013e31815a5fd3, PMID: 18209619

[22] Malkawi H Shannak A Amr S. Surgical treatment of pathological subtrochanteri fractures due to benign lesions in children and adolescents. J Pediatr Orthopaedics. (1984) 4:63–9. doi: 10.1097/01241398-198401000-00014, PMID: 6363449

